# Sleep-Disordered Breathing and Mortality: A Prospective Cohort Study

**DOI:** 10.1371/journal.pmed.1000132

**Published:** 2009-08-18

**Authors:** Naresh M. Punjabi, Brian S. Caffo, James L. Goodwin, Daniel J. Gottlieb, Anne B. Newman, George T. O'Connor, David M. Rapoport, Susan Redline, Helaine E. Resnick, John A. Robbins, Eyal Shahar, Mark L. Unruh, Jonathan M. Samet

**Affiliations:** 1 Johns Hopkins University, Baltimore, Maryland, United States of America; 2 University of Arizona, Tucson, Arizona, United States of America; 3 VA Boston Healthcare System, Boston, Massachusetts, United States of America; 4 University of Pittsburgh, Pittsburgh, Pennsylvania, United States of America; 5 Boston University School of Medicine, Boston, Massachusetts, United States of America; 6 New York University School of Medicine, New York, New York, United States of America; 7 Case Western Reserve University, Cleveland, Ohio, United States of America; 8 American Association of Homes and Services for the Aging, Washington, D. C., United States of America; 9 University of California, Davis, California, United States of America; 10 University of Southern California, Los Angeles, California, United States of America; The George Institute for International Health, Australia

## Abstract

**Background:**

Sleep-disordered breathing is a common condition associated with adverse health outcomes including hypertension and cardiovascular disease. The overall objective of this study was to determine whether sleep-disordered breathing and its sequelae of intermittent hypoxemia and recurrent arousals are associated with mortality in a community sample of adults aged 40 years or older.

**Methods and Findings:**

We prospectively examined whether sleep-disordered breathing was associated with an increased risk of death from any cause in 6,441 men and women participating in the Sleep Heart Health Study. Sleep-disordered breathing was assessed with the apnea–hypopnea index (AHI) based on an in-home polysomnogram. Survival analysis and proportional hazards regression models were used to calculate hazard ratios for mortality after adjusting for age, sex, race, smoking status, body mass index, and prevalent medical conditions. The average follow-up period for the cohort was 8.2 y during which 1,047 participants (587 men and 460 women) died. Compared to those without sleep-disordered breathing (AHI: <5 events/h), the fully adjusted hazard ratios for all-cause mortality in those with mild (AHI: 5.0–14.9 events/h), moderate (AHI: 15.0–29.9 events/h), and severe (AHI: ≥30.0 events/h) sleep-disordered breathing were 0.93 (95% CI: 0.80–1.08), 1.17 (95% CI: 0.97–1.42), and 1.46 (95% CI: 1.14–1.86), respectively. Stratified analyses by sex and age showed that the increased risk of death associated with severe sleep-disordered breathing was statistically significant in men aged 40–70 y (hazard ratio: 2.09; 95% CI: 1.31–3.33). Measures of sleep-related intermittent hypoxemia, but not sleep fragmentation, were independently associated with all-cause mortality. Coronary artery disease–related mortality associated with sleep-disordered breathing showed a pattern of association similar to all-cause mortality.

**Conclusions:**

Sleep-disordered breathing is associated with all-cause mortality and specifically that due to coronary artery disease, particularly in men aged 40–70 y with severe sleep-disordered breathing.

*Please see later in the article for the Editors' Summary*

## Abstract 2

In a cohort of 6,441 volunteers followed over an average of 8.2 years, Naresh Punjabi and colleagues find sleep-disordered breathing to be independently associated with mortality and identify predictive characteristics.

## Editors' Summary

**Background:**

About 1 in 10 women and 1 in 4 men have a chronic condition called sleep-disordered breathing although most are unaware of their problem. Sleep-disordered breathing, which is commonest in middle-aged and elderly people, is characterized by numerous, brief (10 second or so) interruptions of breathing during sleep. These interruptions, which usually occur when relaxation of the upper airway muscles decreases airflow, lower the level of oxygen in the blood and, as a result, affected individuals are frequently aroused from deep sleep as they struggle to breathe. Symptoms of sleep-disordered breathing include loud snoring and daytime sleepiness. Treatments include lifestyle changes such as losing weight (excess fat around the neck increases airway collapse) and smoking cessation. Affected people can also use special devices to prevent them sleeping on their backs, but for severe sleep-disordered breathing, doctors often recommend continuous positive airway pressure (CPAP), a machine that pressurizes the upper airway through a face mask to keep it open.

**Why Was This Study Done?:**

Sleep-disordered breathing is a serious condition. It is associated with several adverse health conditions including coronary artery disease (narrowing of the blood vessels that supply the heart, a condition that can cause a heart attack) and daytime sleepiness that can affect an individual's driving ability. In addition, several clinic- and community-based studies suggest that sleep-disordered sleeping may increase a person's risk of dying. However, because these studies have been small and have often failed to allow for other conditions and characteristics that affect an individual's risk of dying (“confounding factors”), they provide inconsistent or incomplete information about the potential association between sleep-disordered breathing and the risk of death. In this prospective cohort study (part of the Sleep Heart Health Study, which is researching the effects of sleep-disordered breathing on cardiovascular health), the researchers examine whether sleep-disordered breathing is associated with all-cause mortality (death from any cause) in a large community sample of adults. A prospective cohort study is one in which a group of participants is enrolled and then followed forward in time (in this case for several years) to see what happens to them.

**What Did the Researchers Do and Find?:**

At enrollment, the study participants—more than 6,000 people aged 40 years or older, none of whom were being treated for sleep-disordered breathing—had a health examination. Their night-time breathing, sleep patterns, and blood oxygen levels were also assessed and these data used to calculate each participant's apnea-hypopnea index (AHI)—the number of apneas and hypopneas per hour. During the study follow-up period, 1,047 participants died. Compared to participants without sleep-disordered sleeping, participants with severe sleep-disordered breathing (an AHI of ≥30) were about one and a half times as likely to die from any cause after adjustment for potential confounding factors. People with milder sleep-disordered breathing did not have a statistically significant increased risk of dying. After dividing the participants into subgroups according to their age and sex, men aged 40–70 years with severe sleep-disordered breathing had a statistically increased risk of dying from any cause (twice the risk of men of a similar age without sleep-disordered breathing). Finally, death from coronary artery disease was also associated with sleep-disordered breathing in men but not in women.

**What Do These Findings Mean?:**

These findings indicate that sleep-disordered breathing is associated with an increased risk of all-cause mortality, particularly in men aged 40–70 years, even after allowing for known confounding factors. They also suggest that the increased risk of death is specifically associated with coronary artery disease although further studies are needed to confirm this finding because it was based on the analysis of a small subgroup of study participants. Although this study is much larger than previous investigations into the association between sleep-disordered breathing and all-cause mortality, it has several limitations including its reliance on a single night's measurements for the diagnosis of sleep-disordered breathing. Nevertheless, these findings suggest that clinical trials should now be started to assess whether treatment can reduce the increased risk of death that seems to be associated with this common disorder.

**Additional Information:**

Please access these Web sites via the online version of this summary at http://dx.doi.org/10.1371/journal.pmed.1000132.

- The US National Heart Lung and Blood Institute has information (including a video) about sleep-disordered breathing (sleep apnea) (in English and Spanish)
- The UK National Heath Service also provides information for patients about sleep apnea
- MedlinePlus provides links to further information and advice about sleep-disordered breathing (in English and Spanish)
- More information on the Sleep Heart Health Study is available

## Introduction

Sleep-disordered breathing is being increasingly recognized as a cause of substantial morbidity and mortality. Characterized by recurrent collapse of the upper airway, sleep-disordered breathing is associated with recurrent episodes of intermittent hypoxemia and arousals from sleep. Approximately 9% of women and 24% of men in the general population have sleep-disordered breathing and a majority of those affected remain undiagnosed [1]. In addition to causing excessive daytime sleepiness and impaired quality of life [2], sleep-disordered breathing has been implicated in increasing all-cause and cause-specific mortality. Evidence from clinic-based studies suggests that patients with sleep-disordered breathing have a higher mortality risk and that treatment with positive airway pressure during sleep may attenuate this risk [3]–[19]. However, previous studies based on clinical samples have yielded inconsistent results, possibly due to methodological limitations including small sample sizes and use of select patient samples. Furthermore, some of the earlier studies failed to adequately consider potential confounding by obesity and factors such as prevalent hypertension and cardiovascular disease. Recent data from two population-based cohort studies with modest sample sizes have shown that even after accounting for such confounders, sleep-disordered breathing is independently associated with all-cause mortality [20],[21].

The effects of sleep-disordered breathing on mortality may be mediated in part by its association with other medical conditions including hypertension, coronary artery disease (CAD), congestive heart failure, and stroke [22]. Despite the increased burden of adverse health outcomes, epidemiologic information on all-cause and cause-specific mortality in sleep-disordered breathing is limited and uncertainty remains regarding effect modification by other factors. For example, it is not known whether the association between sleep-disordered breathing and mortality varies with age and sex. In addition, little is known about the role of intermittent hypoxemia and sleep disruption, the two characteristic features of sleep-disordered breathing, in mediating the association between sleep-disordered breathing and mortality. Previous work from the Sleep Heart Health Study has shown that sleep-related intermittent hypoxemia is associated with prevalent hypertension, CAD, heart failure, and stroke [23]–[25]. Thus, the degree of intermittent hypoxemia and sleep disruption may also predict all-cause and cause-specific mortality. In the current report, we present longitudinal data collected by the Sleep Heart Health Study, a study of the cardiovascular consequences of sleep-disordered breathing, and examine whether this chronic condition is independently associated with mortality and evaluate the possible effects of sex and age on this association. Secondary objectives included assessment of whether the degree of sleep-related intermittent hypoxemia and frequency of arousals in sleep-disordered breathing are associated with an increased risk of mortality.

## Methods

### Study Design and Population

The Sleep Heart Health Study is a prospective cohort study of cardiovascular consequences of sleep-disordered breathing. Details of the study design have been reported previously [26]. Briefly, between 1995 and 1998 participants were recruited from prospective cohort studies including the Framingham Offspring and Omni Study, the Atherosclerosis Risk in Communities Study, the Cardiovascular Health Study, the Strong Heart Study, and the cohort studies of respiratory disease in Tucson and of hypertension in New York. Eligible individuals were at least 40 years of age and were not being treated for sleep-disordered breathing with positive airway pressure, oral appliance, oxygen, or tracheostomy. A total of 6,441 participants completed the baseline examination and constitute the analysis sample for this report. Each participant in the Sleep Heart Health Study provided written consent and the study protocol was approved by the institutional review board of each participating field site.

### Data Collection

Each participant completed a baseline examination that included a detailed health interview, full-montage unattended home polysomnogram, measurements of blood pressure and anthropometry, as well as assessments of sleep habits and prescription medication use. Prevalent cardiovascular disease was defined as history of physician-diagnosed angina, heart failure, myocardial infarction, stroke, and coronary revascularization, and was determined by adjudicated surveillance data provided by the parent cohorts or by self-report at enrollment. Information on covariates, such as smoking, was obtained by self-report. Anthropometric measures including weight, height, neck circumference, and waist girth were obtained along with three measurements of resting blood pressure during the night of the sleep study by trained and certified technicians.

The sleep study was conducted using a portable monitor (P-Series, Compumedics, Abbotsville, AU). The following signals were recorded: C_3_/A_1_ and C_4_/A_2_ electroencephalograms, bilateral electrooculograms, a single bipolar electrocardiogram, a chin electromyogram, oxyhemoglobin saturation by pulse oximetry, chest and abdominal excursion by inductance plethysmography, airflow by an oronasal thermocouple, and body position by a mercury gauge. Details of polysomnographic equipment, hook-up procedures, failure rates, scoring, and quality assurance and control have been published [27]. Apneas were identified if airflow was absent or nearly absent for at least 10 s. Hypopneas were identified when there was at least 30% reduction in airflow or thoracoabdominal movement for at least 10 s. Apneas were further classified as obstructive if movement on either the chest or abdominal inductance channels was noted, or as central if no displacement was observed on both of these channels. No attempt was made to classify hypopneas as obstructive or central. The apnea–hypopnea index (AHI) was defined as the number of apneas and hypopneas, each associated with a 4% or greater decrease in oxygen saturation, per hour of sleep. An arousal index was defined as the average number of arousals per hour of sleep according to standard criteria [28]. Additional metrics of sleep-disordered breathing severity derived from the sleep study included the percentage of total sleep time with oxyhemoglobin saturation below 90% (TST_90_) and the central apnea index (i.e., the number of central apneas per hour of sleep).

Deaths from any cause, the primary endpoint for this report, were identified and confirmed for the cohort using multiple concurrent approaches including follow-up interviews, written annual questionnaires or telephone contacts with study participants or next-of-kin, surveillance of local hospital records and community obituaries, and linkage with the Social Security Administration Death Master File. Using these methods, 1,047 deaths were identified in the incident cohort with a censoring date of April 1, 2006. During the follow-up period, 147 participants reported treatment with positive airway pressure, an oral appliance, supplemental oxygen, or an open tracheostomy. Sensitivity analyses with exclusion of these participants showed that estimates of mortality risk associated with sleep-disordered breathing remained materially unchanged. Thus, analyses that exclude these participants are reported herein.

### Statistical Analysis

Mortality rates were calculated by dividing number of deaths by number of person–years at risk accumulated. The AHI was categorized using commonly used clinical cutoff points: <5 (normal), 5.0–14.9 (mild disease), 15.0–29.9 (moderate disease), and ≥30.0 events/h (severe disease). In addition, quartile-based cutoff points were also used to categorize the AHI. Sensitivity analyses showed that inferences regarding the association between sleep-disordered breathing severity and mortality were similar regardless of whether clinical or quartile-based thresholds of AHI were used. Thus, the AHI was modeled as a categorical variable using the above clinical cutoff points. Other indicators of sleep-disordered breathing severity (e.g., arousal frequency) were grouped into quartiles. Because TST_90_ and the central apnea index were heavily skewed, each variable was dichotomized using the 75^th^ percentile as the cutoff point. In addition, continuous forms of TST_90_ and the central apnea index were also examined as predictors of mortality.

Kaplan-Meier plots were used to evaluate the association between sleep-disordered breathing severity and mortality. Proportional hazards regression models were then constructed to calculate unadjusted as well as adjusted relative hazard ratios for mortality. Age, sex, race, smoking status, body mass index (BMI), and waist girth were considered as covariates individually and in combination. Age and BMI were modeled as linear terms in the primary models. Quadratic or categorical terms for age and BMI were also examined and were found to not alter model fit or significantly change the parameter estimates for the AHI. To account for potential confounding from preexisting medical conditions, prevalent hypertension, blood pressure, cardiovascular disease (angina, heart failure, myocardial infarction, stroke, and coronary revascularization), diabetes, and smoking status (current, former, or never) at enrollment were included as covariates. Because severity of sleep-disordered breathing and mortality varied significantly as a function of sex and age, additional analyses examined interactions by testing the cross-product terms between AHI, age, and sex. Stratified models were then constructed to examine differential effects of sleep-disordered breathing on mortality by sex and age. In the final stratified models, age was dichotomized as ≤70 and >70 y. The cutoff point of 70 y was based on sensitivity analyses that showed a statistically significant interaction between sleep-disordered breathing severity and age. Finally, the dose–response relationship between AHI and mortality was examined with regression spline analyses. All analyses were conducted using SAS 9.0 (SAS Institute, Cary, NC) and the R statistical package (http://www.r-project.org).

## Results

The analysis cohort included 6,294 participants (53.3% women), which excluded 147 who reported treatment with positive airway pressure, an oral appliance, supplemental oxygen, or an open tracheostomy after the baseline visit. As expected, older age, male sex, minority race, BMI, and central adiposity were associated with increasing severity of sleep-disordered breathing (Table 1). Among men, 42.9% did not have sleep-disordered breathing, 33.2% had mild disease, 15.7% had moderate disease, and 8.2% had severe disease. In women, the corresponding percentages were 64.7%, 24.5%, 7.9%, and 3.0%, respectively. Prevalent hypertension, diabetes, and cardiovascular disease were more common in individuals with moderate to severe sleep-disordered breathing than those with mild or no sleep-disordered breathing. In total, the cohort accumulated 51,523 person–years of observation with an average follow-up duration of 8.2 y. Of the analysis cohort, 1,047 participants (460 women) died during follow-up, yielding a crude mortality rate of 20.3 deaths per 1,000 person-years (95% CI: 19.1–21.6). Men had a higher mortality rate than women (24.8 versus 16.5 per 1,000 person-years, *p*<0.0001; χ^2^ = 42.3) despite similar age and BMI distributions. Mortality rates per 1,000 person–years in the full cohort varied with the AHI category as follows: no sleep-disordered breathing (16.8; 95% CI: 15.4–18.4), mild disease (21.7; 95% CI: 19.4–24.2), moderate disease (28.3; 95% CI: 24.3–33.0), and severe disease (32.2; 95% CI: 26.0–39.8).

**Table 1:** Baseline characteristics, mortality, and follow-up times of the Sleep Heart Health Study cohort by apnea-hypopnea index (AHI) category.

Category	Subcategory	All Participants *N* = 6,294	AHI<5.0 *N* = 3,429	AHI: 5.0–14.9 *N* = 1,797	AHI: 15.0–29.9 *N* = 727	AHI≥30.0 *N* = 341
**Age, years**		62.9 (11.0)	61.3 (11.1)	64.8 (10.6)	65.1 (10.5)	64.6 (10.7)
**BMI, kg/m^2^**		28.4 (5.3)	27.0 (4.5)	29.5 (5.3)	30.7 (5.8)	32.1 (6.1)
**Waist girth, cm**		98.1 (16.7)	93.9 (15.3)	101.0 (13.0)	105.3 (18.6)	109.7 (25.7)
**Neck circumference, cm**		37.9 (4.2)	36.5 (3.9)	38.9 (3.9)	40.1 (4.1)	41.2 (4.1)
**Sex, %**	Women	53.3	63.2	45.7	36.5	29.0
	Men	46.7	36.8	54.3	63.5	71.0
**Race,%**	White	76.6	77.5	76.4	74.4	73.0
	African-American	8.1	8.3	7.1	8.5	10.0
	Native American	9.5	7.7	11.2	12.4	12.0
	Hispanic	4.4	4.9	3.8	4.1	4.1
	Other	1.4	1.6	1.5	0.6	0.9
**Smoking Status, %**	Never	46.0	48.0	43.7	44.5	41.9
	Former	42.6	38.3	47.7	45.8	52.2
	Current	11.4	13.7	8.6	9.7	5.9
**Hypertension, %**		52.4	46.5	57.9	60.0	66.2
**Diabetes, %**		10.6	8.0	12.4	15.6	16.9
**Cardiovascular disease** a **, %**		18.4	14.1	21.7	25.8	28.4
**Deaths, %**		16.6	13.9	17.8	22.7	25.2
**Follow-up time, person-years**		51,523	28,326	14,703	5,823	2,670
**Mortality rate, per 1,000 person-years**		20.3	16.8	21.7	28.3	32.2

*p*<0.0001 for comparisons of age, BMI, waist girth, neck circumference, gender, race, smoking status, hypertension, diabetes, and cardiovascular disease across AHI categories using χ^2^ and analysis of variance to compare categorical and continuous variables, respectively. Values represent mean (standard deviation) or percentage.

a Cardiovascular disease defined as presence of angina, heart failure, myocardial infarction, stroke, or any coronary revascularization procedure.

**Figure 1:**
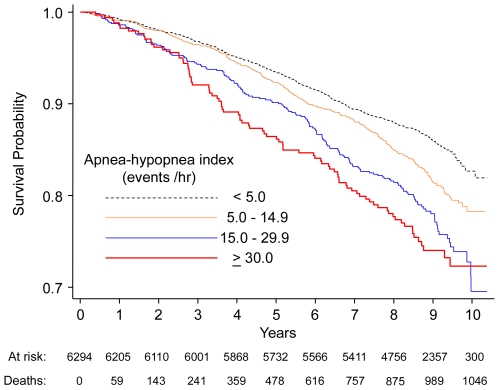
Kaplan-Meier survival curves across categories of the apnea–hypopnea index (AHI).

Figure 1 shows the Kaplan-Meier survival curves across the four AHI categories. Because sleep-disordered breathing severity was different in men and women, multivariable proportional hazards regression models were constructed initially for the full sample and then stratified by sex (Table 2). Three nested models were used to assess incremental effects of various confounding covariates on the association between the AHI and mortality. The base model included AHI as a categorical variable with adjustments for age, sex, and race. The second model added BMI to the base model. The third model added smoking status and prevalent medical conditions as the final set of covariates. Analysis using the full cohort revealed a positive and independent association between sleep-disordered breathing and mortality. Compared to the reference group without sleep-disordered breathing, the fully adjusted hazard ratios for mild, moderate, and severe disease were 0.93 (95% CI: 0.80–1.08), 1.17 (95% CI: 0.97–1.42), and 1.46 (95% CI: 1.14–1.85), respectively. Inclusion of cholesterol levels as an additional covariate did not change the hazard ratios relating sleep-disordered breathing to mortality. Stratified analyses by sex showed that AHI was associated with mortality in men but not women (Table 2).

**Table 2:** Adjusted hazard ratios (95% confidence intervals) for all-cause mortality associated with sleep-disordered breathing in the Sleep Heart Health Study.

Apnea–Hypopnea Index (Events/h)	*N*	Person–Years	Deaths	Mortality Rate^a^	Model 1^b^	Model 2^c^	Model 3^d^
All participants^e^							
<5.0	3,429	28,326	477	16.8	1.00	1.00	1.00
5.0–14.9	1,797	14,703	319	21.7	0.90 (0.78–1.04)	0.93 (0.80–1.07)	0.93 (0.80–1.08)
15.0–29.9	727	5,823	165	28.3	1.16 (0.97–1.39)	1.20 (1.00–1.44)	1.17 (0.97–1.42)
≥30.0	341	2,670	86	32.2	1.30 (1.03–1.64)	1.38 (1.08–1.75)	1.46 (1.14–1.86)
Men							
<5.0	1,262	10,275	216	21.0	1.00	1.00	1.00
5.0–14.9	976	7,873	193	24.5	0.94 (0.78–1.15)	0.99 (0.81–1.20)	1.01 (0.83–1.24)
15.0–29.9	462	3,651	114	31.2	1.23 (0.98–1.54)	1.30 (1.03–1.64)	1.27 (1.00–1.65)
≥30.0	242	1,872	64	34.2	1.30 (0.98–1.72)	1.42 (1.06–1.90)	1.54 (1.15–2.08)
Women							
<5.0	2,167	18,050	261	14.5	1.00	1.00	1.00
5.0–14.9	821	6,830	126	18.5	0.84 (0.68–1.04)	0.85 (0.68–1.06)	0.83 (0.66–1.04)
15.0–29.9	265	2,171	51	23.5	1.05 (0.77–1.42)	1.06 (0.78–1.43)	1.01 (0.73–1.38)
≥30.0	99	798	22	27.6	1.34 (0.86–2.07)	1.37 (0.88–2.13)	1.40 (0.89–2.22)

a Crude mortality rate per 1,000 person-years.

b Model 1: Adjusted for age (continuous) and race.

c Model 2: Adjusted for covariates of model 1 and body mass index (continuous).

d Model 3: Adjusted for covariates of model 2, smoking status (never, former, current), systolic and diastolic blood pressure, prevalent hypertension, diabetes, and cardiovascular disease.

e Sex was included as a covariate in each of the three models based on all participants.

**Figure 2:**
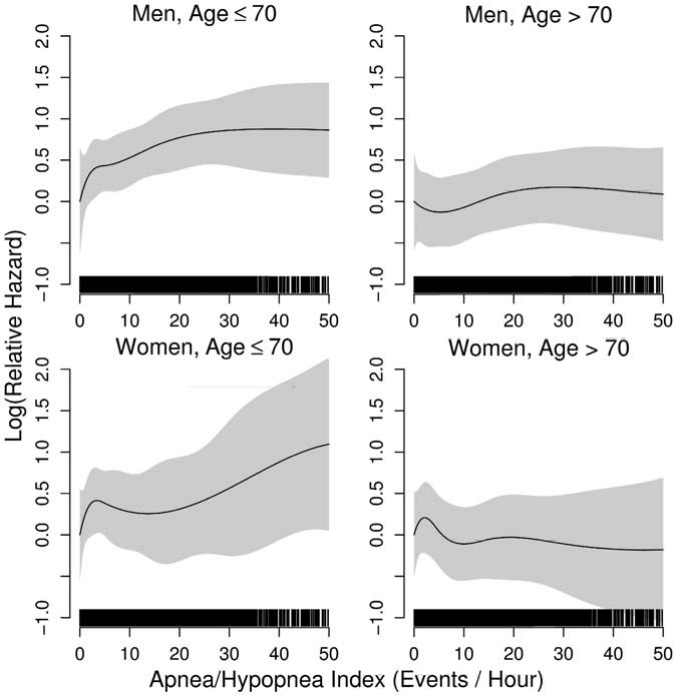
Spline regression models relating the apnea–hypopnea index (AHI) to the log(relative hazard) for all-cause mortality in men and women stratified by age (≤70 and >70 y).

Across all multivariable models, age had a substantial impact on mortality. The two-way interaction between AHI and age was statistically significant (*p*<0.005; χ^2^ = 8.0). In the fully adjusted model for men younger than 70 years, the hazard ratios for mild, moderate, and severe sleep-disordered breathing were 1.24 (95% CI: 0.90–1.71), 1.45 (95% CI: 0.98–2.14), and 2.09 (95% CI: 1.31–3.33), respectively (Table 3). In contrast, sleep-disordered breathing was not associated with mortality in men over 70 years of age. For women, sleep-disordered breathing severity was not associated with mortality in either age group (Table 3). Interestingly, age and sex-stratified spline regression models showed that the mortality risk in men aged 40–70 y increased linearly from relatively low AHI values without evidence of a specific threshold for excess risk (Figure 2). The lack of a significant association in the younger women is likely due to the limited numbers of participants with severe disease.

**Table 3:** Adjusted hazard ratios (95% confidence intervals) for all-cause mortality associated with sleep-disordered breathing,stratified by sex and age in the Sleep Heart Health Study.

Apnea–Hypopnea Index (Events/h)	*N*	Person–Years	Deaths	Mortality Rate^a^	Model 1^b^	Model 2^c^	Model 3^d^
Men ≤70 y							
<5.0	985	8,220	91	11.1	1.00	1.00	1.00
5.0–14.9	694	5,697	82	14.4	1.10 (0.81–1.48)	1.16 (0.85–1.58)	1.24 (0.90–1.71)
15.0–29.9	322	2,623	47	17.9	1.37 (0.96–1.95)	1.44 (1.00–2.08)	1.45 (0.98–2.14)
≥30.0	168	1,355	28	20.7	1.67 (1.09–2.55)	1.88 (1.19–2.95)	2.09 (1.31–3.33)
Men >70 y							
<5.0	277	2,055	125	60.8	1.00	1.00	1.00
5.0–14.9	282	2,176	111	51.0	0.86 (0.67–1.11)	0.89 (0.69–1.16)	0.92 (0.70–1.20)
15.0–29.9	140	1,029	67	65.1	1.18 (0.87–1.58)	1.25 (0.92–1.70)	1.23 (0.90–1.68)
≥30.0	74	517	36	69.6	1.16 (0.80–1.69)	1.25 (0.85–1.83)	1.27 (0.86–1.86)
Women ≤70 y							
<5.0	1641	13,902	90	6.5	1.00	1.00	1.00
5.0–14.9	527	4,448	40	9.0	1.00 (0.68–1.45)	0.99 (0.66–1.47)	0.97 (0.64–1.48)
15.0–29.9	161	1,350	14	10.4	1.11 (0.63–1.96)	1.12 (0.62–2.02)	1.15 (0.63–2.11)
≥30.0	63	537	8	14.9	1.73 (0.84–3.58)	1.75 (0.82–3.74)	1.76 (0.77–3.95)
Women >70 y							
<5.0	526	4,149	171	41.2	1.00	1.00	1.00
5.0–14.9	294	2,382	86	36.1	0.77 (0.60–1.00)	0.78 (0.60–1.02)	0.77 (0.58–1.00)
15.0–29.9	104	821	37	45.1	0.98 (0.68–1.40)	0.99 (0.69–1.42)	0.89 (0.61–1.31)
≥30.0	36	261	14	53.6	1.09 (0.62–1.89)	1.10 (0.63–1.92)	1.14 (0.65–2.01)

a Crude mortality rate per 1,000 person years.

b Model 1: Adjusted for age (continuous) and race.

c Model 2: Adjusted for covariates of model 1 and body mass index (continuous).

d Model 3: Adjusted for covariates of model 2, smoking status (never, former, current), systolic and diastolic blood pressure, prevalent hypertension, diabetes, and cardiovascular disease.

Analyses were undertaken to assess whether the arousal frequency, the central apnea index, and measures of sleep-related hypoxemia (i.e., TST_90_) were associated with mortality. Regardless of age and other covariates, arousal frequency and the central apnea index (Tables 4 and 5) were not associated with mortality in men or women irrespective of whether these measures were modeled continuously or categorically. However, TST_90_ was a significant predictor of mortality in men less than 70 y in age, even after adjusting for age, race, smoking status, BMI, systolic and diastolic blood pressure, AHI, prevalent hypertension, diabetes, and cardiovascular disease. Compared to the first three quartiles (TST_90_≤2.70%), younger men in the fourth quartile (TST_90_>2.70%) had an adjusted hazard ratio of 1.83 (95% CI: 1.31–2.52) for mortality. In older men and women of both age categories, TST_90_ was not associated with mortality.

**Table 4:** Adjusted hazard ratios (95% confidence intervals) for the association between quartiles of arousal frequency and all-cause mortality in the Sleep Heart Health Study.

Arousal Frequency (Events/h)	Model 1^a^	Model 2^b^	Model 3^c^
All participants^d^			
<12.0	1.00	1.00	1.00
12.0–16.7	0.84 (0.70–1.02)	0.84 (0.70–1.05)	0.86 (0.70–1.05)
16.8–23.5	0.98 (0.82–1.17)	0.98 (0.82–1.18)	0.97 (0.80–1.17)
≥23.5	0.98 (0.82–1.16)	0.99 (0.83–1.18)	0.98 (0.82–1.17)
Men			
<13.7	1.00	1.00	1.00
13.7–18.7	0.99 (0.77–1.27)	1.00 (0.78–1.29)	1.00 (0.77–1.29)
18.8–25.8	1.11 (0.87–1.41)	1.12 (0.88–1.43)	1.17 (0.91–1.50)
≥25.9	1.11 (0.88–1.41)	1.15 (0.91–1.46)	1.14 (0.89–1.45)
Women			
<10.8	1.00	1.00	1.00
10.9–15.0	0.84 (0.63–1.12)	0.84 (0.63–1.13)	0.88 (0.66–1.19)
15.1–21.2	0.93 (0.71–1.22)	0.94 (0.72–1.23)	0.89 (0.67–1.17)
≥21.3	0.88 (0.68–1.14)	0.88 (0.68–1.15)	0.90 (0.68–1.17)

a Model 1: Adjusted for age (continuous) and race.

b Model 2: Adjusted for covariates of model 1 and body mass index (continuous).

c Model 3: Adjusted for covariates of model 2 and smoking status (never, former, current), hypertension, diabetes, and cardiovascular disease.

d Sex was included as a covariate in each of the three models based on all participants.

**Table 5:** Adjusted hazard ratios (95% confidence intervals) for the association between the central apnea index and all-cause mortality stratified by sex and age.

Central Apnea Index (Events/h)	Model 1^a^	Model 2^b^	Model 3^c^
All participants^d^			
<0.26	1.00	1.00	1.00
≥0.26	0.99 (0.85–1.16)	0.99 (0.84–1.15)	1.00 (0.86–1.18)
Men			
<0.44	1.00	1.00	1.00
≥0.44	1.15 (0.95–1.41)	1.15 (0.95–1.41)	1.16 (0.94–1.42)
Women			
<0.16	1.00	1.00	1.00
≥0.16	1.10 (0.87–1.40)	1.10 (0.87–1.41)	1.08 (0.84–1.39)

a Model 1: Adjusted for age (continuous) and race. Hazard ratios were derived comparing the lower three quartiles to the fourth quartile of the central apnea index.

b Model 2: Adjusted for covariates of model 1 (except sex) and body mass index (continuous).

c Model 3: Adjusted for covariates of model 2 and smoking status (never, former, current), hypertension, diabetes, and cardiovascular disease.

d Sex was included as a covariate in each of the three models based on all participants.

The association between sleep-disordered breathing and CAD-specific mortality was further examined in the 220 deaths that occurred during follow-up. As before, sex-stratified multivariable analyses were undertaken. However, given the limited number of events, further stratification by age was not possible. In men, an AHI≥15 events/h had a fully adjusted hazard ratio of 1.69 (95% CI: 1.13–2.52) for CAD-related death. In women, an association was not identified between sleep-disordered breathing and CAD-related deaths.

## Discussion

Using a prospective cohort study, which included middle-aged and older adults from several United States communities, we examined the independent association between sleep-disordered breathing and mortality. The results of this study demonstrate that, independent of several confounding variables, sleep-disordered breathing was associated with all-cause and cardiovascular disease–related mortality. The association was most apparent in men aged 40–70 y with severe disease (AHI≥30 events/h). The degree of sleep-related hypoxemia was found to be independently associated with mortality, whereas the arousal frequency and the central sleep apnea index were not.

The question of whether sleep-disordered breathing decreases survival has great clinical and public health significance. To date, a number of studies on clinical populations have been carried out on the association between sleep-disordered breathing and mortality and the findings have been conflicting [3]–[13]. Discrepancies across the available clinic-based studies have resulted, in part, from inconsistencies in methods used to assess breathing abnormalities during sleep, differences in disease definition, and variable attention to potential confounding factors. In one of the earliest case series of 1,620 patients with sleep-disordered breathing, Lavie et al. [10] showed that an apnea index of more than 30 events/h was associated with all-cause mortality in young and middle-aged men. While this study highlighted the importance of sleep-disordered breathing as a risk factor for mortality, its findings are limited by the exclusion of women, the lack of consideration of treatment effects, and the concern for referral bias that is inherent in clinical populations. Although a subsequent report, which included 14,589 male patients from the same clinical center, confirmed the earlier observation that sleep-disordered breathing is independently associated with mortality, all of the methodological concerns remained [17].

Several groups have also examined the association of sleep-disordered breathing and mortality in community or population cohorts. Unfortunately, even in these studies, the use of snoring as a surrogate for sleep-disordered breathing [12] or the assessment of only elderly participants [11] limits inferences regarding the association between sleep-disordered breathing and mortality for the general population. Recent findings from the Wisconsin Sleep Cohort and the Busselton Sleep Cohort studies, which have incorporated objective measures of sleep-disordered breathing in general population samples, show an independent association between sleep-disordered breathing and all-cause mortality [20],[21]. In the Wisconsin study, the fully adjusted hazard ratio for all-cause mortality comparing people with severe disease to those without disease (AHI≥30 events/h versus. <5 events/h) was 2.7 (95% CI: 1.3–5.7). Cardiovascular disease-related mortality in the Wisconsin study was also higher in people with severe disease than those without disease (hazard ratio: 5.2; 95% CI: 1.4–19.2). While the Busselton study did not examine cause-specific mortality, it too found that moderate-to-severe sleep-disordered breathing was associated with all-cause mortality with an adjusted hazard ratio of 6.2 (95% CI: 2.0–19.4). Although results from both of these studies are congruent with each other and with the results presented herein, the relatively small number of participants with moderate-to-severe sleep-disordered breathing and the limited number of deaths in either cohort (33 in the Busselton cohort and 80 in the Wisconsin cohort) limited the ability to assess effect modification by age and sex and characterize dose–response associations between sleep-disordered breathing and mortality. Furthermore, the potential associations between sleep-related oxyhemoglobin desaturation, recurrent arousals from sleep, occurrence of central sleep apneas, and mortality were not assessed by either study.

The Sleep Heart Health Study adds to the available evidence by demonstrating that sleep-disordered breathing is associated with mortality and that this association is potentially mediated by the degree of sleep-related intermittent hypoxemia. The association with mortality was observed in only those participants with an AHI≥30 events/h. The current study also finds that excess mortality is most apparent in men aged ≤70 y. Although a similar association was also observed in younger women, particularly with severe disease (AHI≥30 events/h), the adjusted hazard ratios did not reach statistical significance. Because of the limited number of deaths in younger women with moderate to severe disease in our study, we cannot exclude an independent association between sleep-disordered breathing and mortality in women. Additional research is needed to determine whether a longer period of follow-up with a sufficient number of diseased people would uncover whether sleep-disordered breathing is independently related to mortality in women. Furthermore, the negative finding in older adults (age >70 y) should not obviate the clinical concern for identifying and treating sleep-disordered breathing in this subgroup. With increasing age, the likelihood of death from other causes rises so that quantifying the potential association between sleep-disordered breathing and mortality becomes more difficult. It is also possible that, compared to younger or middle-aged adults, sleep-disordered breathing in the older adults may be distinct in its impact on clinical outcomes.

Several potential mechanisms could underlie the higher mortality risk in sleep-disordered breathing. A number of studies over the last decade, including the Sleep Heart Health Study [23],[24], have shown that sleep-disordered breathing is associated with hypertension, CAD, congestive heart failure, and stroke [22]. Sleep-disordered breathing has also been implicated as a risk factor for insulin resistance and type 2 diabetes mellitus [29]. Although many of these consequences have been documented in cross-sectional analyses, several lines of evidence indicate that sleep-disordered breathing may be a causal factor. Perhaps the most persuasive evidence is that treatment with positive airway pressure improves blood pressure [30],[31] and may decrease cardiovascular events [18]. In addition, there are data suggesting that sleep-disordered breathing can shift the temporal vulnerability for sudden cardiac death [32]. In the absence of sleep-disordered breathing, the greatest risk for sudden cardiac death is between 6 a.m. and 11 a.m. However, in patients with sleep-disordered breathing, more than half of sudden cardiac deaths occur between 10 p.m. and 6 a.m., suggesting that the associated hemodynamic and physiologic stress may trigger malignant arrhythmias. Finally, in addition to cardiovascular mortality, noncardiovascular causes of death may also contribute to excess mortality, given that people with sleep-disordered breathing are more prone to motor vehicle accidents particularly those associated with personal injury [33].

There are several limitations of the current study that merit discussion. First, because cause-specific death was only adjudicated for cardiovascular disease, hypothesis testing regarding mortality from non-cardiovascular causes was not possible. Second, measurement error from short-term variability in sleep and breathing patterns might have diluted the mortality risk, as only one night of recording was used to assess disease severity. However, previous work on night-to-night variability on sleep and breathing patterns has shown that one night of recording provides a reasonably accurate estimate of sleep-disordered breathing severity [34]. Third, a number of the potentially confounding covariates (e.g., smoking status) were obtained by self-report. Nonetheless, in all probability, any bias in self-reported data is likely to be unrelated to the abnormalities on the sleep study and thus would not lead to biased estimates of mortality risk. Fourth, because the Sleep Heart Health Study participants were recruited from ongoing epidemiological studies of cardiovascular and respiratory disease, survival bias may have contributed to some of our findings on sleep-disordered breathing and mortality, particularly in older participants. Finally, it is important to recognize that the threshold used to present the age-stratified results (≤70 versus >70 y) was based on sensitivity analyses that examined several different age cutoff points. Multiple examinations of any data can lead to identification of statistically significant findings just based on chance. Thus, while the Sleep Heart Health Study data indicate a differential relation between sleep-disordered breathing and mortality in younger and older participants, the age threshold of 70 years should not be overinterpreted. These limitations notwithstanding, the current study also has several strengths including the longitudinal assessment of a large community sample with careful characterization of sleep and breathing abnormalities, comprehensive assessment of key covariates and outcomes, and analytic consideration of effect modification by age and sex.

In conclusion, the Sleep Heart Health Study shows that sleep-disordered breathing is an independent predictor of mortality and that this association is not attributable to age, obesity, or other chronic medical conditions. Although the degree of nocturnal hypoxemia was an independent predictor of mortality, arousal frequency and occurrence of central apneas were not. Given the high and likely increasing prevalence of sleep-disordered breathing in the general population, additional research in the form of randomized clinical trials should be undertaken to assess if treatment can reduce premature mortality associated with this common and chronic disorder.

## Abbreviations

AHI: apnea–hypopnea index
BMI: body mass index
CAD: coronary artery disease
TST_90_: total sleep time with oxyhemoglobin saturation below 90%
